# Editorial Notes, News and Miscellany

**Published:** 1914-05

**Authors:** 


### Editoiral Notes, News and Miscellany.

The Rockefeller Institute for Medical Research has recently received an additional endowment from John D. Rockefeller of one million dollars.

---

The American Proctologic Society will hold its sixteenth annual meeting in Atlantic City, N. J., on June 22 and 23, 1914. An interesting program has been arranged. Headquarters will be at the Hotel Chalfonte.

---

Mrs. Hortense Ward's article in the Woman's Department this month is a valuable contribution dealing with the status of married women under our present laws.

---

The American Society for the Control of Cancer held a most profitable public meeting in St. Louis on May first. Such eminent men as Frederick L. Hoffman, William F. Rodman, Leo Loeb and Curtis E. Lakeman contributed to the programme of helpful addresses.

---

At the meeting of the American Surgical Association Dr. William J. Mayo stated that the prevalence of cancer is perhaps due to present methods of cooking. He asserted that cancer of the stomach forms a third of all the cancer cases among civilized people, while among the lower animals cancer of the stomach does not form nearly so large a proportion of the cases.

---

THE FIRST TOUR of the American Society for Physicians' Study will be made immediately after the coming meeting of the American Medical Association, starting on June 26 from Atlantic City. The complete itinerary may be obtained from the secretary, Dr. Albert Bernheim, 1225 Spruce Street, Philadelphia. Early bookings through the secretary are urged, in order that satisfactory accommodations may be secured for all who desire to participate.

---

MOST of the cities of Texas are waging an early and relentless war on the fly. Swatting the fly does not suffice to destroy the pest. but the removal of the breeding places is necessary. Filthy barns and stables, with their accumulations of filth, are the principal sources of supply. Where it is impossible to remove the accumulations from the barn immediately, the following method assists in destroying the flies, and should be used even where the most prompt removal of stable manure is practiced: In five gallons of water put two tablespoonfuls of creosote and a pint of of kerosene oil. With this mixture spray every few days the manure piles, stalls and places where accumulations of trash have been. In this way the eggs will be destroyed, and if the spraying is continued there will be marked decrease in the number of flies.

---

THE MASSACHUSETTS LEGISLATURE gives promise of passing some constructive health measures, the public health committees having favorably reported on the following measures: "That the State Board of Health supervise and direct work of local boards of health; that the State Board of Health shall have authority to make rules for ventilation of schoolhouses; that no male patient above the age of fourteen shall be sent to the Westfield Hospital for Consumptives; that the State Board of Health may request a judge of a police, district or municipal court to order the removal of a tuberculosis patient from his home to some proper hospital; that the State Board of Health shall have a department of diseases dangerous to the public health; that cities and towns may borrow outside of their debt limits to erect hospitals for tuberculosis; and that the trustees of the hospital for consumptives may provide treatment for incorrigible tuberculosis patients."

